# Cephalosporins as key lead generation beta-lactam antibiotics

**DOI:** 10.1007/s00253-022-12272-8

**Published:** 2022-11-19

**Authors:** Xuemei Lin, Ulrich Kück

**Affiliations:** Allgemeine und Molekulare Botanik, Fakultät für Biologie und Biotechnologie, Ruhr-Universität Bochum, 44780 Bochum, Germany

**Keywords:** Cephalosporins, Beta-lactam antibiotics, Antibiotic resistance, 7-ACA, Bacterial acylase, Key lead generation

## Abstract

**Abstract:**

Antibiotics are antibacterial compounds that interfere with bacterial growth, without harming the infected eukaryotic host. Among the clinical agents, beta-lactams play a major role in treating infected humans and animals. However, the ever-increasing antibiotic resistance crisis is forcing the pharmaceutical industry to search for new antibacterial drugs to combat a range of current and potential multi-resistant bacterial pathogens. In this review, we provide an overview of the development, innovation, and current status of therapeutic applications for beta-lactams with a focus on semi-synthetic cephalosporins. Cephalosporin C (CPC), which is a natural secondary metabolite from the filamentous fungus *Acremonium chrysogenum*, plays a major and demanding role in both producing modern antibiotics and developing new ones. CPC serves as a core compound for producing semi-synthetic cephalosporins that can control infections with different resistance mechanisms. We therefore summarize our latest knowledge about the CPC biosynthetic pathway and its regulation in the fungal host. Finally, we describe how CPC serves as a key lead generation source for the in vitro and better, in vivo synthesis of 7-aminocephalosporanic acid (7-ACA), the major core compound for the pharmaceutical synthesis of current and future semi-synthetic cephalosporins.

**Key points:**

**•** *Latest literature on cephalosporin generations*

**•** *Biotechnical production of cephalosporins*

**•** *In vivo production of 7-ACA*

## Introduction

The modern medicinal era is basically marked by the discovery of penicillin in 1928 by Alexander Fleming in London, a significant milestone that saved countless human lives from lethal infectious diseases (Fleming 1929). The large-scale production of penicillin was achieved in the 1940s by the pharmaceutical company Eli Lilly in the USA, and penicillin was soon introduced into clinical therapy as the first beta-lactam antibiotic (Madison 1989). This class of antibiotics comprises a collective group of medicinal compounds possessing antibacterial activity based on a common mechanism, namely, disrupting bacterial cell wall construction, which is facilitated by the beta-lactam ring of the core molecular structure.

Following the successful discovery of penicillin, three other main classes of beta-lactam antibiotics based on natural secondary metabolites were developed over the last century. These were cephalosporins and carbapenems, comprising a bicyclic nucleus resembling penicillin’s core chemical structure, discovered in 1948 and 1976, and subsequently in 1981, monobactam antibiotics, which represent a unique class of beta-lactam antibiotics characterized by a monocyclic system (Brotzu 1948; Brown et al. 1976; Imada et al. 1981; Newton and Abraham 1954; Sykes et al. 1981). Their distinct core structure as well as various side chain modifications determine their variable spectrum of bactericidal activities, pharmacological properties, and the particular extent of tolerance towards antibiotic resistance.

*Penicillins* are one of the most important groups of antibiotics, with high efficacy and low toxicity; their core structure comprises a five-membered thiazolidine ring attached to the beta-lactam ring. Natural penicillins (penicillin G and penicillin V) and narrow-spectrum penicillins (methicillin, nafcillin, oxacillin, dicloxacillin) are most active against Gram-positive organisms, while the broad-spectrum penicillins (amoxicillin, ampicillin) are active against both Gram-positive and Gram-negative bacteria (Raj 2021). Bacterial beta-lactamases, a group of enzymes capable of disrupting the beta-lactam ring structure by hydrolysis, are the most frequently reported source of penicillin antibiotic resistance, especially to natural penicillins (Raj 2021). Therefore, semi-synthetic penicillins (methicillin, nafcillin, oxacillin, dicloxacillin), which are resistant to beta-lactamases, were developed to preserve the efficacy and avoid the restricted application of penicillins.

*Cephalosporins* are alternative beta-lactam antibiotics, which are highly effective and commonly used for mild to severe infectious diseases. Recently, prescription numbers of cephalosporins in the USA (47.5% of total antibiotics) exceeded the number of narrow- and wide-spectrum penicillins (39.7% of total antibiotics) as recorded by the healthcare database from 2004 to 2014 mentioned above (Bush and Bradford 2016). Semi-synthetic cephalosporins are broadly active against both Gram-positive and Gram-negative bacteria, and they are synthesized based on the bicyclic nucleus consisting of a six-membered dihydrothiazine ring attached to a beta-lactam ring. Two carbons of the cephalosporin scaffold, C3 and C7, confer a huge possibility for introducing variable side chains that significantly extend antibacterial activities as well as enhance the structural stability against beta-lactamases.

*Carbapenems* possess the broadest spectra of antibiotic activity among all classes of beta-lactam antibiotics (Domachowske and Suryadevara 2020). Imipenem, the first carbapenem available for clinic treatment of complex infections, is a derivative of thienamycin discovered in *Streptomyces cattleya* (Kahan et al. 1979; Moellering et al. 1989). The five-membered pyrroline ring is fused to a beta-lactam ring at the core structure. The stable bonds and saturated atoms in the scaffold increase the resistance against most beta-lactamases (Domachowske and Suryadevara 2020). Therefore, carbapenems were chosen for patients with severe infections caused by antibiotic resistant bacteria. In addition, carbapenems are used as beta-lactamase inhibitors (Papp-Wallace et al. 2011).

*Monobactams* are monocyclic molecules comprising a beta-lactam ring and side chains. The first monobactam launched on the market was Aztreonam, isolated from the bacteria *Chromobacterium violaceum* (Sykes et al. 1981). Monobactams are solely active against Gram-negative bacteria. However, the superior property of monobactams is that they are highly resistant to beta-lactamases produced by Gram-negative bacterium (Raj 2021). Furthermore, no cross-reaction was found between penicillins and cephalosporins; thus, monobactams may not cause allergies in patients who are allergic to other beta-lactam agents (Raj 2021).

As a highly effective group of wide-spectrum antibiotics, beta-lactams are the most dominant anti-infectious agents, leading both the antibiotic market and clinical prescription numbers. A healthcare database reported that 65.24% of total injectable antibiotic consumption in the USA from 2004 to 2014 was represented by beta-lactam antibiotics (Bush and Bradford 2016). Moreover, global research in 79 countries between 2000 and 2015 summarized that more than 60% of total defined daily doses (DDD) of antibiotic consumption were attributed to beta-lactam antibiotics, mostly comprising wide-spectrum penicillins (39% of total DDD) and semi-synthetic cephalosporins (20% of total DDD) (Klein et al. 2018). This research also revealed that the consumption rate of beta-lactam antibiotics is increasing globally, largely driven by low- to middle-income countries (Klein et al. 2018).

Initially, it was believed that widespread antimicrobial resistance was unlikely. However, the excessive use of antibiotics led to the widespread of antimicrobial resistances including beta-lactams and thus became a major threat to human health (Aslam et al. 2018). Major sources for acquiring antimicrobial resistances are the exorbitant use of antibiotics in the treatment of patients, in animal feeding, or through direct or indirect contact to contaminated environment (Kim and Ahn 2022). Therefore, advances in wide areas of pharmaceutical research, including genetic engineering and synthetic chemistry are necessary to develop new antibiotics that may provide effective solutions to treat arising resistances.

In this review, we summarize the importance and great relevance of cephalosporins as key lead generation beta-lactam antibiotics to overcome the increasing antimicrobial resistances in clinical applications. Furthermore, we discuss the current state of biotechnical strategies to produce semi-synthetic cephalosporins.

## Cephalosporin antibiotics

Cephalosporin antibiotics are derived from the filamentous fungus *Acremonium chrysogenum*. Cephalosporin C (CPC) was the first cephalosporin antibiotic compound isolated. Its chemical structure was characterized soon after its discovery by Giuseppe Brotzu from Italy in 1945 (Abraham and Newton 1961; Newton and Abraham 1955). CPC and its derivatives have broad antibacterial activity against Gram-positive and Gram-negative bacteria. They are essential for hospital patients to prevent and treat infectious diseases occurring on the skin, ear, and bone, as well as upper respiratory and urinary tract infections. Over the last decades, various highly effective semi-synthetic cephalosporins have been developed and widely prescribed worldwide.

The following sections provide insights into the characteristic features of each class of cephalosporins, how they evade antibiotic resistance mechanisms, and the latest state of cephalosporin use and development. Moreover, the focus of this review is on the production of cephalosporin antibiotic derivatives, introducing CPC as a core lead generation compound for biosynthesis and industrial production strategies of semi-synthetic cephalosporin building blocks.

Cephalosporins are bactericidal agents inhibiting growth through disrupting the cross-linking of the peptidoglycan chains of bacterial cell walls. The final step for constructing the peptidoglycan layer is catalyzed by penicillin-binding proteins (PBP), which cross-link the linear glycopeptides to form a 3-dimensional structure. As a characteristic feature of beta-lactam antibiotics, cephalosporins bind to PBPs by mimicking the structure of glycopeptides, irreversibly inhibiting bacterial cell wall production (Tipper and Strominger 1965). To date, five major generations of cephalosporin antibiotics have been developed, which are categorized by the timeline of discovery as well as their spectrum of antibiotic activity. The generic and trade names for some representative cephalosporins in each generation are listed in Table 1, and the current status of applications in the USA and the conventional clinical administration routes are indicated.

**Table 1:** Overview of five generations of cephalosporin antibiotics

Generic name	Trade name	FDA approval status		Route of administration
First generation				
** Cefalexin**	Keflex, Ceporex	H		PO
Cefadroxil	Duricef	H, V		PO
Cefazolin	Ancef, Kefzol	H		IM, IV
Cefapirin	Cefadyl	W		IM, IV
Cefradine	Intracef, Velosef	W; OC		PO, IM, IV
Cefalotin	Keflin	W; OC		IV
Second generation				
** Cefaclor**	Ceclor, Keflor, Raniclor	H		PO
Cefprozil	Cefzil, Cefprozil	H		PO
** Cefuroxime**	Zinacef, Ceftin, Kefurox	H		PO, IM, IV
Cefoxitin	Mefoxin	H		IM, IV
Cefotetan	Cefotan	H		IM, IV
Loracarbef	Lorabid	W		PO
Cefonicid	Monocid	W		IM, IV
Cefmetazole	Zefazone	W		IM, IV
Third generation				
** Cefixime**	Suprax	H	PO	
** Cefpodoxime**	Vantin	H, V	PO	
Cefotaxime	Claforan	H	IM, IV	
Ceftriaxone	Rocephin	H	IM, IV	
Ceftazidime*	Avycaz, Tazicef, Fortaz	H	IM, IV	
Cefoperazone*	Cefobid	W	IM, IV	
Ceftizoxime	Cefizox	W	IM, IV	
Fourth generation				
Cefepime*	Maxipime	H	IM, IV	
Cefiderocol*	Fetroja	H	IV	
Cefpirome*	Cefrom	W	IV	
Fifth generation				
Ceftaroline	Teflaro, Zinforo	H	IV	
Ceftolozane*	Zerbaxa	H	IV	
Ceftobiprole*	Zevtera, Mabelio	W; OC	IV	

Bold type indicates the agents that represented the top 90% of the community consumption of cephalosporins in 28 European Union / European Economic Area (EU/EEA) countries in 2017. * The cephalosporins that are active against the multidrug-resistant pathogen *Pseudomonas aeruginosa*. Compiled from (Harrison and Bratcher 2008), (Versporten et al. 2021), and the current information from the US Food and Drug Administration (FDA) (Drugs@FDA 2022). Abbreviations: *PO*: oral administration; *IM*: intramuscular administration; *IV*: intravenousadministration; *H*, agents that have acquired FDA approval for human use; *V*, approved for veterinarian applications; *W*, agents discontinued or withdrawn from the US market due to safety or effectiveness issues. *OC*, still sold in countries other than the USA

### Classification of cephalosporin antibiotics

The first-generation cephalosporins are highly active against Gram-positive cocci, such as streptococci, including *Streptococcus pneumoniae* which is responsible for the most community acquired pneumonia cases, and methicillin-sensitive *Staphylococcus aureus* (MSSA) (Harrison and Bratcher 2008). The first-generation oral cephalosporins are well absorbed and distributed in most tissues except cerebrospinal fluid (CSF) and middle ear fluid. However, first-generation cephalosporins have relatively weak activity against Gram-negative bacteria such as *Pseudomonas aeruginosa* or *Enterobacter*. These are considered multidrug-resistant pathogens, namely, resistant to at least three different classes of antimicrobials.

The limitation of the first-generation cephalosporins was overcome by the introduction of second-generation cephalosporins to the clinic. These cephalosporins showed higher stability against beta-lactamases produced by some Gram-negative bacteria, such as *Haemophilus influenza* (related to respiratory infections) or some species from Enterobacteriaceae (Harrison and Bratcher 2008). The significant advantages of second-generation cephalosporins are the reduced dosage and extended half-life, which benefits the patients administered these antibiotics (Tartaglione and Polk 1985).

Third-generation cephalosporins increased the spectrum of antibiotic activities against Gram-negative bacteria, including *Enterobacter*, beta-lactamase-producing *H. influenzae*, and meningococci (often cause meningitis) (Barriere and Flaherty 1984). Therefore, these highly effective cephalosporins are often used to treat sepsis of unknown origin. Additionally, second-generation and third-generation cephalosporins can be administered to patients allergic to penicillin, since the cross-reaction between first-generation cephalosporins and penicillin antibiotics arises from the similar chemical structures at the side chains but not the beta-lactam ring (Pichichero and Casey 2007). Many of the third- and later-generation cephalosporins can adequately penetrate the CSF and are thus favored for treating central nervous system infections such as meningitis (Sullins and Abdel-Rahman 2013).

Despite their broad antibacterial activity, the effectiveness of third-generation cephalosporins has become limited due to increasing resistance emerging in bacteria, typically within Enterobacteriaceae. For instance, *P. aeruginosa*, a Gram-negative aerobic bacterium, is a highly problematic pathogen related to hospital-acquired severe infections such as ventilator-associated pneumonia and blood infections. As an opportunistic, nosocomial pathogen of immunocompromised individuals, *P. aeruginosa* has gained resistance to an extensive range of antibiotics, which makes treatment extremely challenging. Most third-generation cephalosporins are ineffective in treating *P. aeruginosa* infections, except ceftazidime and cefoperazone. In contrast, fourth-generation cephalosporins all have excellent activity against this bacteria*.*

Overall, fourth-generation cephalosporins have been developed to strongly target a broader range of Gram-negative bacteria. This improved activity results from altering the side chain orientation. This enables rapid penetration of cephalosporins through the outer membrane and reduces the binding affinity with beta-lactamases, thus escaping the resistance present in many Gram-negative bacteria (Garau et al. 1997). Moreover, fourth-generation cephalosporins also show excellent activity against Gram-positive bacteria such as penicillin-resistant pneumococci, some streptococci, and MSSA (Garau et al. 1997). Therefore, hospitalized patients with severe infections have recently been treated with fourth-generation cephalosporins (Wilson 1998); however once again, this posed the problem of selection pressures leading to resistance.

More powerful antibiotics were urgently required under the pressure of exponentially increasing multidrug-resistant pathogens. The fifth-generation cephalosporins, developed for treating multidrug-resistant bacterial strains, possess remarkably extended antibiotic spectra, notably including the methicillin-resistant *Staphylococcus aureus* (MRSA) (Bui and Preuss 2022; Selvan and Ganapathy 2016). In 2019, MRSA was the second leading pathogen causing deaths associated with drug resistance, after the most frequent death-associated pathogen *E. coli* (Murray et al. 2022). MRSA is responsible for community-acquired severe skin and soft tissue infections and necrotizing pneumonia (Gonzalez et al. 2005; Moran et al. 2006). The wide-ranging resistance of MRSA was developed from the frequent exchange of resistant genetic elements encoding mutant PBPs. Those mutants bind to the beta-lactam rings of all other beta-lactam antibiotics, disrupting their target binding actions (Zhanel et al. 2009).

### Resistance mechanisms against cephalosporins

Cephalosporins are often the preferred group of antibiotics due to their broad antibacterial spectrum and fewer side effects than other types of antibiotic drugs. They are highly effective and indispensable drugs used against numerous infectious diseases in humans and animals. However, excessive use and misuse of cephalosporins for prophylaxis, treatment, or food production have significantly contributed to the emergence of many drug-resistant pathogens.

Three primary mechanisms exist for causing antibiotic resistance against cephalosporins (Livermore 1987). (i) Bacteria produce enzymes that inactivate the antibiotic, for example, beta-lactamases. This is the most common and crucial mechanism of resistance in Gram-negative bacteria. Beta-lactamases are bacterial enzymes that can break the beta-lactam ring by hydrolysis. At certain levels of bactericidal pressure, the expression of genes encoding beta-lactamases is regulated and inducible (Dancer 2001). (ii) Resistance also arises from alterations in PBPs, the cephalosporins’ target protein. Bacteria modify their PBPs either by generating mutant variants with significantly lower affinity to the beta-lactam agents or by developing a novel PBP that does not bind to the beta-lactam structure. (iii) Reduced amounts of antibiotics reaching their target results in a drug resistance phenotype. For Gram-negative bacteria, resistance may occur due to the low membrane permeability of antibiotics. Structural or quantitative changes in porin, a bacterial outer membrane protein transporting hydrophilic molecules, can inhibit the penetration of cephalosporins. These porin alterations mainly result from point mutations or downregulated gene expression. In addition, the efflux pump contributes to antibiotic resistance by inhibiting drug diffusion. The energy-driven efflux system expels antibiotics from the periplasmic space of Gram-negative bacteria, where the peptidoglycan layer is located (Poole 2007).

Cephalosporin resistance can be intrinsic or acquired through mobile genetic elements from other bacteria (Navarro 2006). In other words, the genes encoding determinants of cephalosporin resistance are located on bacterial chromosomes, plasmids, transposons, or integrons. Antibacterial resistance can be horizontally transferred between bacteria through conjugation, transformation, and bacteriophage transduction. These mechanisms allow bacteria to exchange and acquire naked DNA from their environment (transformation) or the genetic components from other unrelated species (transduction) (Navarro 2006). Thus, it is not unanticipated to find the resistance elements in water, in animal feces, or even in food products (Chen et al. 2017). These findings highlight the importance to develop new antimicrobials, and recently some new and stronger beta-lactamases received attention. A representative example is the extended-spectrum beta-lactamases (ESBL), which are plasmid-encoded, and able to hydrolyze third-generation cephalosporins (Babic et al. 2006; Paterson and Bonomo 2005). In some cases, two or even more resistance mechanisms are found simultaneously in antibiotic-resistant pathogenic strains. For example, porin loss was found in an ESBL-expressing *E. coli* strain (Martínez-Martínez 2008). *P. aeruginosa* is another typical example. Besides its intrinsic resistance driven by the efflux pumps, it also quickly acquires resistance either by chromosomal gene mutations or horizontal gene transfer, obtaining genes encoding beta-lactamases (Cornelis 2008; Poole 2004). Such complex resistances cause complications in recognizing pathogenic bacterial types in clinical diagnosis and result in limited options for efficient therapeutic antibiotics. Therefore, combinations of an antibiotic and a beta-lactamase inhibitor (e.g., ceftazidime-avibactam or ceftolozane-tazobactam against *P. aeruginosa*) have been suggested to enhance bactericidal action, even in the presence of a certain level of resistance to the antibiotic alone (Zhanel et al. 2013).

### Current state of clinical use and development of cephalosporins

In addition to intensive therapy applications in hospitals, significant amounts of cephalosporins are prescribed to cure infections in the community. According to a study conducted in 28 countries of the European Union/European Economic Area (EU/EEA), in 2017, cephalosporins represented 11.6% of antibiotic consumption in the community (Bruyndonckx et al. 2021). Among all the classes of cephalosporin antibiotics, five compounds dominated the community cephalosporin market, accounting for 90% of total consumption, namely, cefalexin (8.0%) from the first generation; cefuroxime (63.0%) and cefaclor (7.2%) from the second generation; and cefixime (8.9%) and cefpodoxime (3.1%) from the third generation (Table 1) (Versporten et al. 2021). Considerable variations in trends were observed from 1997 to 2017. Proportional consumption of second-generation and third-generation cephalosporins increased significantly over time, whereas that of first-generation cephalosporins gradually decreased. The proportional consumption of fourth-generation cephalosporins is negligible and decreased considerably over time (from 0.02% in 2009 to 0.01% in 2017) (Adriaenssens et al. 2011; Versporten et al. 2021).

As a milestone in modern medicine, the discovery of antibiotics has saved countless lives following medical procedures such as surgery and when treating numerous infectious diseases. However, the improper use of antibiotics threatens our health system and ability to save lives. In Europe, about 33,000 people die every year due to infections caused by antibiotic-resistant bacteria (Cassini et al. 2019). Approximately 30% of infants with sepsis die due to antibiotic resistance (Laxminarayan et al. 2016). In 2016, the World Health Organization (WHO) published a list of the world’s leading antibiotic-resistant bacteria, for which new treatments are urgently needed (Tacconelli et al. 2018). The critical risk pathogens from this list are *Pseudomonas* spp. and Enterobacteriaceae, which have developed resistance to the extremely powerful antibiotic carbapenem. In addition to carbapenem-resistant Enterobacteriaceae (CRE), the vancomycin-resistant enterococci (VRE) and MRSA pathogens were prioritized for enhanced surveillance, control, and research activities investigating new active ingredients.

Along with the rapidly escalating antibiotic-resistant crisis, the successful discovery of a new class of antibiotic is particularly challenging. From 1987 to the early 2010s, no significant patent or advance was achieved for a novel class of antibiotics (Silver 2011). An active substance is considered innovative when it has a new target or mechanism of action, without cross-resistance within existing antibiotics of the same class. Another criterion of significant success in drug research and development is the very high specificity of an active ingredient to the bacterial target to minimize toxic effects in patients (Silver 2011). However, many compounds under development fail, due to low selectivity or resistance exhibited by the pathogens tested.

**Fig. 1:**
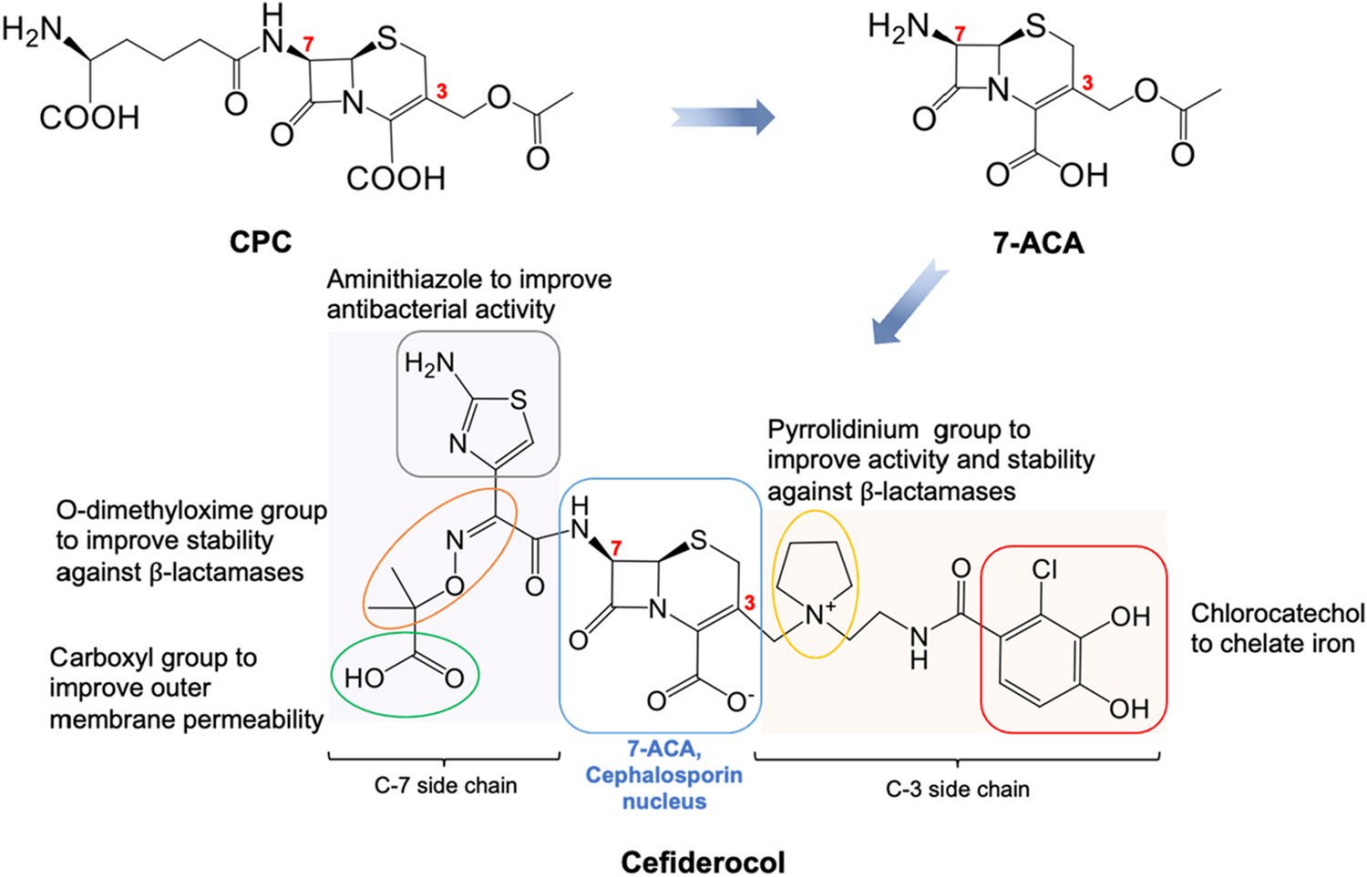
The chemical structure of cefiderocol, a novel class of cephalosporins. 7-ACA, the core moiety to construct semi-synthetic cephalosporin antibiotics, is produced from CPC, followed by a series of chemical modifications to add functional groups on the 3′ and 7′ positions of the cephalosporin nucleus. The distinct and essential functional groups of cefiderocol are marked, and the roles contributing to its superior antibacterial activity are noted. Modified from Terreni et al. (2021)

Cefiderocol, an outstanding agent from a novel class of cephalosporins, has overcome all these hindrances in antibiotics development. It was approved by the FDA in 2019 and by European Medicines Agency (EMA) in 2020, under the trade name Fetroja (Terreni et al. 2021). Cefiderocol is the first siderophore cephalosporin, where a chlorocatechol moiety chelates iron to form the iron-cefiderocol antibiotic complex, leading to efficient transport into bacterial cells through iron transporters (Fig. 1). In addition, the quaternary ammonium from the pyrrolidinium group supports the penetration as a zwitterion; also, the carboxylic acid from the side chain increases the permeability through the outer membrane. Hydrolysis of cefiderocol by beta-lactamase (including ESBL and carbapenemase) is protected by the O-dimethyloxime and pyrrolidinium groups. Due to these structural advantages, cefiderocol could overcome most antibiotic resistance mechanisms. Moreover, its efficacy and safety have been approved. Thus, cefiderocol is considered a promising agent for treating severe or life-threatening infections caused by Enterobacteriaceae, *P. aeruginosa*,* H. influenzae*, and* Acinetobacter baumannii*, i.e., the multidrug-resistant bacteria and beta-lactamase-producing bacteria with limited treatment options or no alternative therapy (Terreni et al. 2021; Zhanel et al. 2019).

## Cephalosporin C biosynthesis in *A. chrysogenum*

### Cephalosporin C biosynthetic pathway

**Fig. 2:**
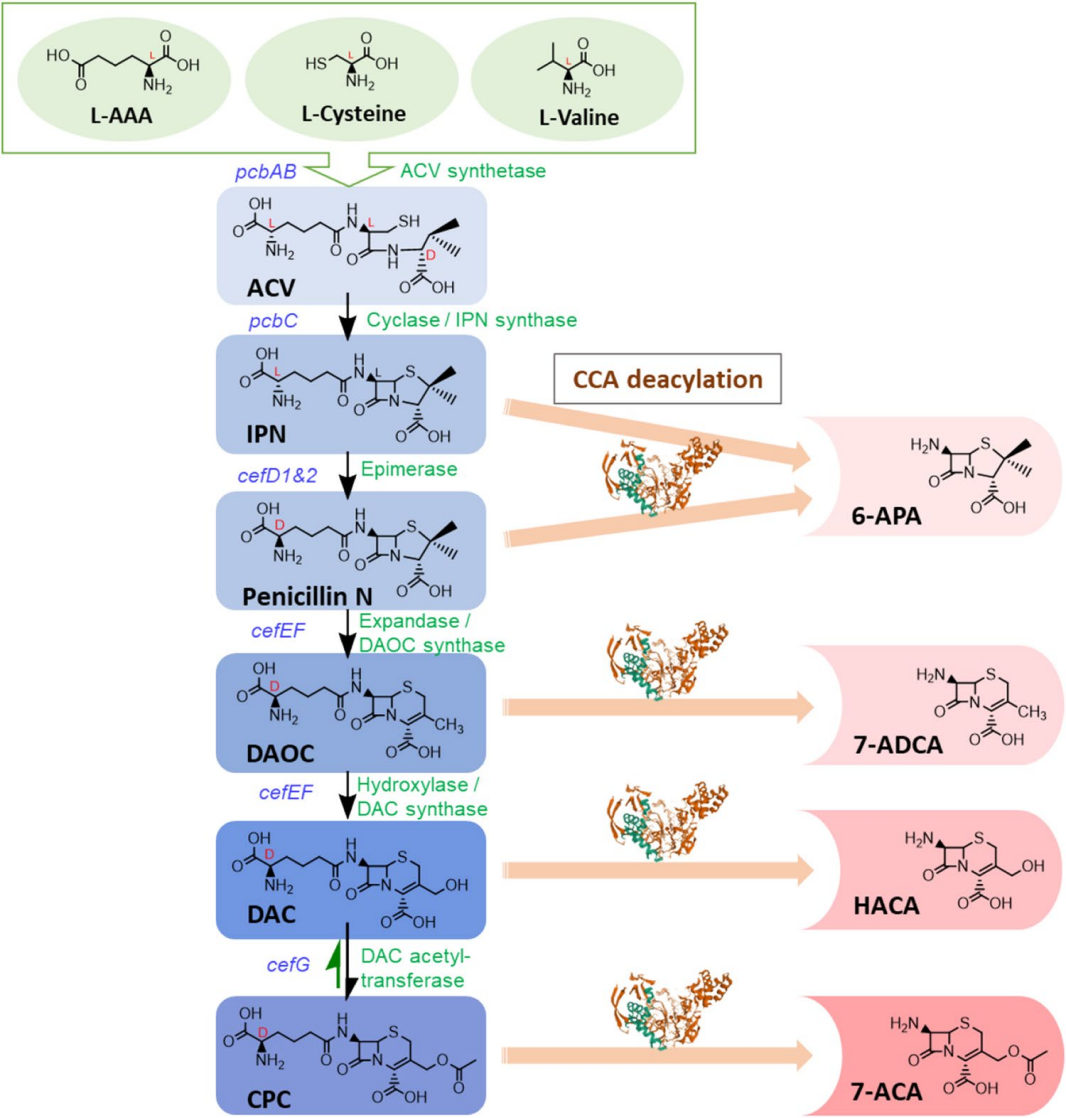
Cephalosporin C biosynthetic pathway in *A. chrysogenum* and the conversion products of each metabolite by cephalosporin C acylase. Biosynthetic enzymes are indicated in green, and their encoding genes are written in blue. The cephalosporin C acylase (CCA) protein structure was obtained from the Protein Data Bank entry: 1FM2 (Kim et al. 2000), modified based on Schmitt et al. (2004b)

Cephalosporin C (CPC) is exclusively produced by the filamentous fungus *A. chrysogenum.* The entire biosynthetic pathway has been studied in detail due to its high pharmaceutical and economic value. In pharmaceutical manufacturing companies, classical strain improvement is routinely performed to maintain high production lines with huge CPC yields. Recently, 24–28 g/l CPC yields were achieved from fed-batch fungal fermentation on a production scale (Meyer et al. 2016). Recently, highly developed molecular biotechnology has enabled the detection and quantification of each intermediate. Moreover, the catalytic enzymes involved in CPC biosynthesis and transport are characterized, and their genes have been thoroughly investigated (Fig. 2) (Schmitt et al. 2004b).

Like penicillin and other beta-lactam antibiotics, CPC biosynthesis is initiated by three amino acids, L-cysteine, L-valine, and the non-proteinogenic L-α-aminoadipic acid (L-α-AAA). These three amino acids are catalyzed to form the tripeptide ACV by the ACV-synthetase in the cytosol. Cyclization of the ACV molecule is mediated by isopenicillin N (IPN) synthase. From here on, the biosynthesis pathways differ for different groups of beta-lactam antibiotics. In cephalosporin biosynthesis, the resulting IPN is isomerized to penicillin N through the two-component epimerases located in peroxisomes. The subsequent reaction occurs in the cytosol again, where penicillin N is converted to deacetoxycephalosporin C (DAOC) by DAOC synthetase. Here, the five-membered thiazolidine ring attached to the beta-lactam ring is expanded to the six-membered dihydrothiazine ring, which is the distinctive structure of cephalosporins. DAOC side chain modification facilitated by a cytoplasmic hydroxylase follows, generating deacetylcephalosporin C (DAC). Finally, acetyltransferase adds an acetyl-CoA (coenzyme A) group to DAC to produce CPC. CPC is actively secreted from the fungal cytosol to the extracellular space as a natural antibacterial substance. However, the transport mechanism is still unclear, although the *cefT* gene, encoding a putative efflux pump, was reported to be involved in CPC export (Ullan et al. 2002).

### Regulation of CPC biosynthesis

Since the 1990s, genomic, transcriptomic, and proteomic research into the regulatory mechanisms of CPC biosynthesis has been driven by advanced molecular biological tools and growing bioinformatic data. *A. chrysogenum* genes encoding CPC biosynthetic enzymes have been investigated by many research groups at their mRNA levels in different phases of fermentation, with or without additional supplements, or under different growth media conditions. Several studies indicated that various regulatory factors such as methionine, glucose, and ambient pH alter transcriptional levels of biosynthetic genes, initiating the regulation of CPC biosynthesis (Jekosch and Kück 2000; Schmitt et al. 2001; Velasco et al. 1994).

Several transcription factors are responsible for activating or repressing one or multiple genes, resulting in further significant changes in CPC productivity. Velvet is a global regulatory protein for filamentous fungi, first discovered in *Aspergillus nidulans*. AcVEA is the homolog of velvet found in *A. chrysogenum*, which regulates CPC biosynthesis by controlling the transcription of *cefEF* and is also involved in hyphal fragmentation (Dreyer et al. 2007). A recent transcriptomic analysis revealed that 35–57% of the velvet target genes showed altered expression in improved *A. chrysogenum* and *P. chrysogenum* industrial strains, demonstrating that the increase in secondary metabolite production via strain improvement programs is mainly due to regulatory changes rather than mutations in gene sequences (Terfehr et al. 2017).

PacC is a zinc finger transcription factor regulating the expression of many genes in a pH-dependent manner in *A. chrysogenum* and other fungi such as *Penicillium chrysogenum*,* A. nidulans*, and *Fusarium oxysporum* (Caracuel et al. 2003; Espeso et al. 1997; Suárez and Peñalva 1996). Schmitt and her colleagues identified PacC binding domains at promoter regions of *pcbAB-pcbC* and *cefG*-*cefEF* genes of *A. chrysogenum* (Schmitt et al. 2001). CRE1 is a repressive transcription factor responding to the carbon source glucose, putatively binding at *pcbC* and *cefEF* genes (Velasco et al. 1994). CPCR1 is another transcription factor binding at the *pcbC* gene, thus regulating an early phase in the CPC biosynthesis pathway (Schmitt et al. 2004a).

In addition, a regulatory impact can be due to enzyme activity without transcriptional modification. Such regulations directly change the performance of the CPC biosynthesis enzymes. For example, ACV synthetase is inhibited by the presence of glucose and the carbon source metabolite glyceraldehyde-3-phosphate (G3P) (Zhang et al. 1989). Alternatively, some regulations may have an indirect influence on enzymes relevant to CPC biosynthesis. For instance, the activity of L-α-AAA reductase from lysine biosynthesis is significantly reduced in CPC high production strains to ensure an adequate supply of the energy source ATP and L-α-AAA, both essential starting materials for CPC biosynthesis (Zhang and Demain 1992). The plasma membrane H^+^-ATPase (PMA) is another example that modulates the ATP level in fungal cells, thus regulating the energy supply for CPC biosynthesis (Hijarrubia et al. 2002; Zhgun et al. 2020).

In filamentous fungi, the biosynthesis of secondary metabolites is often associated with cell development and differentiation. Secondary metabolites are commonly produced within the development stage of spore formation (Calvo et al. 2002). During strain development processes, high-yield *A. chrysogenum* production strains lost the function for conidiospore development. However, a special differentiation type of *A. chrysogenum* is the swollen fragment or yeast-like cell actively producing CPC, namely, the arthrospore (Bartoshevich et al. 1990). The high productivity of yeast-like mycelial fragments might rely on a cytochrome-independent alternative respiratory pathway, whereas the correlation between mycelial fragmentation and the CPC production rate is not particularly strict (Karaffa et al. 1996; Sándor et al. 1998). Nevertheless, enhanced hyphal fragmentation is often accompanied by upregulated CPC biosynthesis stimulated by different regulatory factors, e.g., methionine, CPCR1, or velvet (Dreyer et al. 2007; Hoff et al. 2005; Karaffa et al. 1997).

## Chemoenzymatic conversion from CPC to 7-ACA

**Fig. 3:**
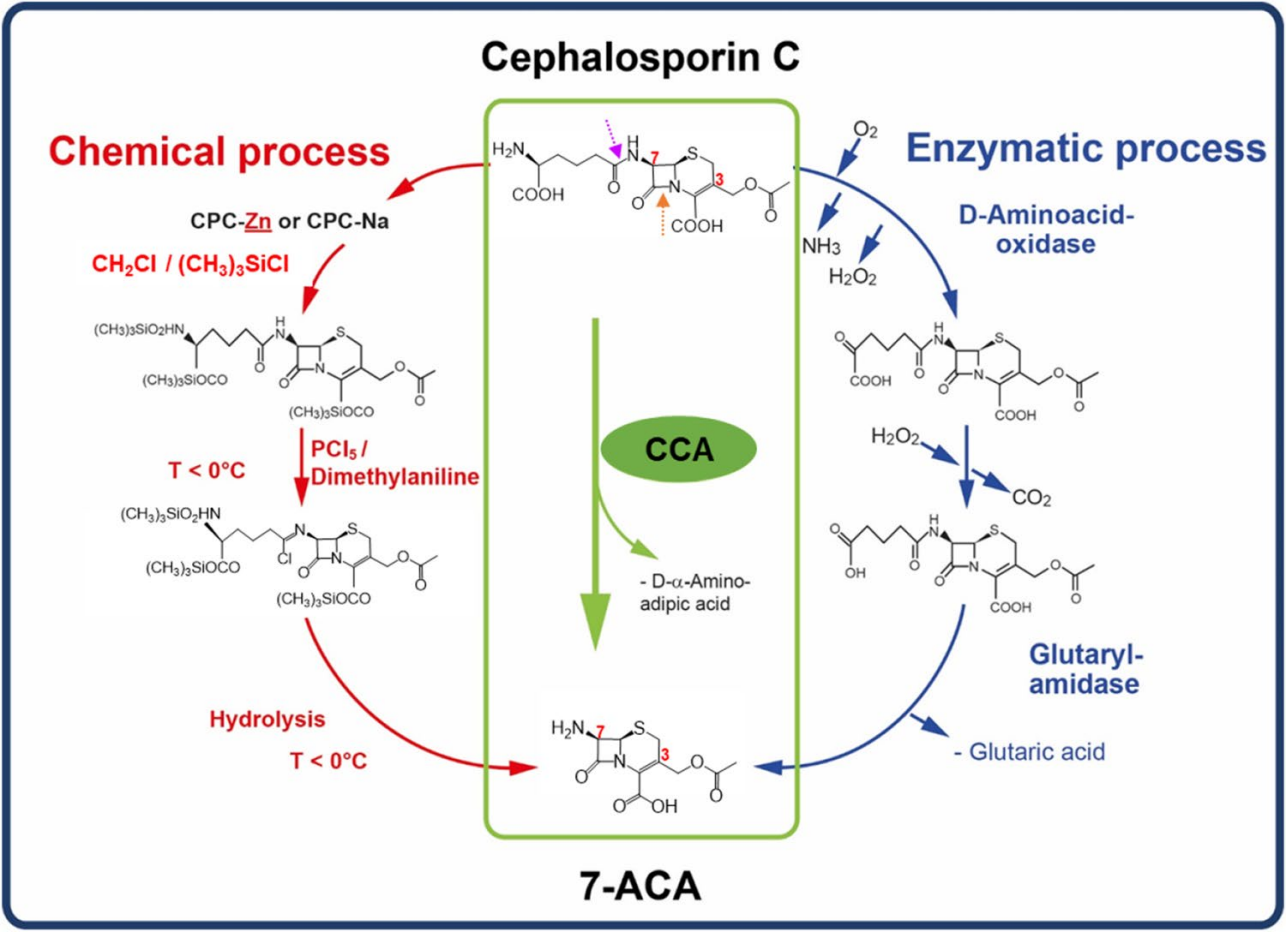
The conversion of cephalosporin C to 7-ACA. On the left the chemical process, and on the right the enzymatic manufacturing processes currently applied in the pharmaceutical industry. Achieving one-step bioconversion using cephalosporin C acylase (CCA) is the subject of this study, as highlighted in the middle. C-3 and C-7 are indicated in 7-ACA, the two carbon sites subjected to different side chain modifications to synthesize various cephalosporin antibiotics. At the top, the magenta arrow indicates the acyclic amide bond, and the orange arrow indicates the amide bond in the beta-lactam ring. Modified from Groeger et al. (2017)

7-Aminocephalosporanic acid (7-ACA) is the fundamental substrate of most of the semi-synthetic cephalosporins. Some cephalosporins, such as oral cefadroxil and cefalexin from the first-generation cephalosporins, are derived from 7-amino-deacetoxycephalosporanic acid (7-ADCA). These effective cephalosporins are chemically derived by various side chain modifications on the C-3 and C-7 positions of 7-ACA, establishing broad and robust antibacterial activity and high chemical stability (Fig. 3).

Originally, 7-ACA was synthesized chemically from CPC using a series of reactions (Fig. 3) (Cabri 2008; Huber et al. 1972). Of the two amide bonds in the CPC structure, one is a more stable acyclic amide on the side chain attached to the beta-lactam ring, and the other is a less stable and more reactive cyclic amide within the beta-lactam ring. The desired chemical reaction from CPC to 7-ACA is hydrolysis of the more stable side chain acyclic amide bond while keeping the beta-lactam structure intact. Thus, not only it is a highly challenging process conducted at extremely low temperatures using organic solvents, but also this chemical production method caused severe environmental harm, i.e., releasing toxic chemical waste and polluted water containing phosphates (Cabri 2008; Morin et al. 1962).

Later, a two-step enzymatic procedure was developed with a yield similar to chemical production and eventually substituted the classical chemical synthesis of 7-ACA (Fig. 3) (Riethorst and Reichert 1999). Here, the first enzyme reaction uses the D-amino acid oxidase (DAO) produced by wild-type *Trigonopsis variabilis* as a whole-cell biocatalyst. This modifies the 7-aminoadipyl side chain of CPC to generate glutaryl-7-aminocephalosporanic acid (GL-7-ACA). The second enzyme is the GL-7-ACA acylase which converts GL-7-ACA to the final product 7-ACA. GL-7-ACA acylase is a heterologous recombinant protein produced and isolated from *E. coli*, and applied in vitro as an immobilized biocatalyst (Riethorst and Reichert 1999).

Notably, the waste released from the two-step enzymatic procedure was drastically reduced to 1% of the chemical method (Cabri 2008; Groeger et al. 2017; Wohlgemuth 2010). Therefore, compared to the chemical process, this two-step enzymatic method is environmentally more sustainable, and as a second benefit more economically attractive. The immobilized DAO in the stirred-tank bioreactor and the purified GL-7-ACA acylase could be recycled up to 100 and 600 reaction cycles. Also, only mild temperature and pH reaction conditions are required. However, this two-step enzymatic procedure to produce 7-ACA requires two different bacterial fermentation procedures and complicated enzyme purification-immobilization processes. Therefore, manufacturing costs for 7-ACA and the active pharmaceutical ingredients of cephalosporins were still relatively high.

## Cephalosporin C acylase

A one-step bioconversion is now the state-of-the-art technology applied in industrial 7-ACA production. The single enzyme required here is the cephalosporin C acylase (CCA) (Fig. 3). CCAs have been discovered in various bacterial strains and are categorized into five classes based on gene similarity, protein size, and enzyme properties (Table 2) (Aramori et al. 1991; Pollegioni et al. 2013). CCA molecules comprise an α-subunit, a spacer, and a β-subunit from the N- to C-terminus; some CCAs contain a signal peptide before the α-subunit. CCA is a member of the N-terminal nucleophile hydrolase superfamily, which also includes glutamine phosphoribosylpyrophosphate amidotransferase (GPAT), penicillin acylase, and the proteasome (Brannigan et al. 1995). The acylase reaction is catalyzed by Ser1β, the N-terminal amino acid of the β-subunit. This residue is also critical for the enzyme’s activity as a nucleophile and the proton donor.

**Table 2:** Classification of cephalosporin C acylases. Modified from Tan et al. (2018) and Pollegioni et al. (2013)

Class	Source	Strain name	Molecular weight (kDa)	Subunit structure (kDa)	Signal peptide (aa)	Spacer (aa)	Relative activity^a^	
							**To GL-7-ACA**	**To CPC**
I	*Pseudomonas* sp.	KAC-1 (130)^b^	70	16 + 54	29	10	100%	2.3%
	*Pseudomonas* sp.	SY-77 (GK-16)^b^	70	16 + 54	27	10	100%	0
II	*Pseudomonas* sp.	A14	89	28 + 61	29	-	100%	0
III	*Pseudomonas diminuta*	N176	84	26 + 58	-	10	100%	4%
	*Pseudomonas* sp.	SE 83 (AcyII)^c^	83	26 + 57	23	10	100%	5%
	*Pseudomonas diminuta*	V22	84	26 + 58	-	-	100%	3.2%
IV	*Pseudomonas* sp.	SE 83 (AcyI)^c^	62	40 + 22	-	-	100%	0
V	*Bacillus laterospous*	J1	70	Monomeric	27	-	100%	0

^a^Relative activity of acylases on GL-7-ACA was taken as 100%

^b^This acylase was called two different names in literature

^c^Two different acylases were identified from *Pseudomonas* sp. SE83

CCAs are synthesized as inactive precursor polypeptides and then undergo a post-translational enzymatic activation process. The molecular mechanism is well characterized via site-directed mutagenesis and protein mass spectrometry analysis (Kim and Kim 2001; Lee and Park 1998; Li et al. 1999). The maturation of CCA requires a two-step autocatalytic process to form the active αβ heterodimer (Lee and Park 1998). The first step is an intramolecular cleavage at the C-terminal end of the spacer, generating the β-subunit and the α-subunit peptide including the spacer. As mentioned above, the nucleophilic serine at the N-terminus of the resulting β-subunit catalyzes this hydrolytic cleavage. Also, the glycine at the C-terminus of the spacer plays a vital role due to its carbonyl group (Li et al. 1999). The second autoproteolytic modification is an intermolecular cleavage removing the 10 amino acid spacer from the C-terminus of the α-subunit. Here, the Ser1β again plays an essential role as a nucleophile, and the carboxylate group of the glutamic acid or aspartic acid at the N-terminus of the spacer is critical as it can mimic the substrate GL-7-ACA and bind to the active site pocket of CCA (Kim and Kim 2001). A previous study demonstrated that the individual α-subunit and β-subunit are inactive, but they can recover acylase activity when co-expressed in *E. coli* (Li et al. 1999). For some CCAs containing a signal peptide connected to the α-subunit, the effect of removing the signal peptide is negligible on CCA precursor processing and enzyme activity; the same conclusion was reached for changing several amino acids at the N-terminus of the α-subunit (Li et al. 1999).

The challenge of applying CCA in industrial production is that CCA takes GL-7-ACA as a primary substrate and shows low activity with CPC. In the last two decades, many studies have been performed on the engineering potential of CCAs. Some acylases or glutaryl amidases were modified to efficiently cleave the acyclic amide bond in CPC (Kim and Hol 2001; Kim et al. 2000; Oh et al. 2003; Otten et al. 2004; Pollegioni et al. 2013). Kim and colleagues revealed the first crystal structure of class I CCA at 2.0 Å resolution (Kim et al. 2000). They found that the αβ heterodimer forms a bowl-like structure, consisting of α-helices at the outer surface and 19 β-strands inside the bowl. The conformational deacylation mechanism catalyzed by the nucleophile Ser1β was elucidated through this protein 3D structure. The critical residues in the substrate-binding pocket were also identified; eight were from the β-subunit, and only two were from the α-subunit. Since then, various molecular modeling and site-directed mutagenesis approaches have been performed to improve the substrate specificity of CCAs (Cho et al. 2009; Kim and Kim 2001).

Pharmaceutical companies have used highly active CCAs for one-step conversion since 2006, applied as an immobilized biocatalyst produced by *E. coli* (Groeger et al. 2017; Zhu et al. 2011). Until now, the industrial application of CCA is limited to the enzyme process conducted in vitro. Recently, a promising in vivo approach to convert CPC into 7-ACA was reported (Lin et al. 2022). These authors constructed a bacterial acylase gene, whose genetic codon usage was adapted to the fungal host, to successfully express the gene in the CPC producing fungus *A. chrysogenum*. Mass spectrometry analysis showed that the bacterial enzyme is processed successfully in the fungal cell, and the active enzyme is secreted into the culture supernatant. Using optimized fermentation-incubation conditions, the researchers observed a 30% conversion rate from CPC to 7-ACA. It is to be anticipated that transfer of this laboratory approach to industrial production lines will require adaptation to large manufacturing scales and facilities. Nevertheless, this straightforward approach minimizes sophisticated manufacturing processes, and would be ecologically and economically beneficial for sustainable antibiotic production.

## Conclusion

Cephalosporin antibiotics are widely prescribed for various bacterial infections in the community and, more importantly, for severe infectious diseases in hospitals caused by antibiotic-resistance bacteria. Most semi-synthetic cephalosporins are derived from the core building block 7-ACA by chemically modifying side chains. Here, *A. chrysogenum* plays a crucial role as the industrial producer of CPC, the substrate for the enzymatic production of 7-ACA. Therefore, investigating the CPC-producing fungus *A. chrysogenum* and novel 7-ACA production methods is highly attractive for industrial and economic reasons.

Discovering environmentally harmless and sustainable manufacturing processes would undoubtedly benefit future pharmaceutical processes. The industry has constantly been seeking solutions for lowing manufacturing costs. For that, industrial companies have accomplished maximizing CPC yields from fungal fermentation via routinely performed *A. chrysogenum* strain improvements. Meanwhile, another approach to reducing manufacturing costs is minimizing the enzymatic processes after the fermentation steps. One-step direct conversion from CPC to 7-ACA has been achieved in vitro using CCA on an industrial scale (Groeger et al. 2017). Introducing such a one-step bioconversion system into the CPC-producing fungus is an attractive approach to upgrade further innovative production. Once the CCA enzyme is expressed and activated near its substrate biosynthesis site, CPC can immediately be converted into 7-ACA, thus eliminating entire enzyme-related downstream processes and significantly reducing manufacturing costs.

## References

[CR1] Abraham EP, Newton GG (1961). The structure of cephalosporin C. Biochem J.

[CR2] Adriaenssens N, Coenen S, Versporten A, Muller A, Minalu G, Faes C, Vankerckhoven V, Aerts M, Hens N, Molenberghs G (2011). European surveillance of antimicrobial consumption (ESAC): outpatient quinolone use in Europe (1997–2009). J Antimicrob Chemother.

[CR3] Aramori I, Fukagawa M, Tsumura M, Iwami M, Isogai T, Ono H, Ishitani Y, Kojo H, Kohsaka M, Ueda Y (1991). Cloning and nucleotide sequencing of new glutaryl-7-ACA and cephalosporin C acylase genes from *Pseudomonas* strains. J Ferment Bioeng.

[CR4] Aslam B, Wang W, Arshad MI, Khurshid M, Muzammil S, Rasool MH, Nisar MA, Alvi RF, Aslam MA, Qamar MU, Salamat MKF, Baloch Z (2018). Antibiotic resistance: a rundown of a global crisis. Infect Drug Resist.

[CR5] Babic M, Hujer AM, Bonomo RA (2006). What's new in antibiotic resistance? Focus on beta-lactamases. Drug Resist Updat.

[CR6] Barriere SL, Flaherty JF (1984). Third-generation cephalosporins: a critical evaluation. Clin Pharm.

[CR7] Bartoshevich YE, Zaslavskaya P, Novak M, Yudina O (1990). *Acremonium*
*chrysogenum* differentiation and biosynthesis of cephalosporin. J Basic Microbiol.

[CR8] Brannigan JA, Dodson G, Duggleby HJ, Moody PC, Smith JL, Tomchick DR, Murzin AG (1995). A protein catalytic framework with an N-terminal nucleophile is capable of self-activation. Nature.

[CR9] Brotzu G (1948) Research on a new antibiotic. Publications of the Cagliari Institute of Hygiene 5–19. http://medicina.unica.it/pacs/brotzuen.pdf

[CR10] Brown AG, Butterworth D, Cole M, Hanscomb G, Hood JD, Reading C, Rolinson GN (1976). Naturally-occurring beta-lactamase inhibitors with antibacterial activity. J Antibiot.

[CR11] Bruyndonckx R, Adriaenssens N, Versporten A, Hens N, Monnet DL, Molenberghs G, Goossens H, Weist K, Coenen S (2021). Consumption of antibiotics in the community, European Union/European Economic Area, 1997–2017. J Antimicrob Chemother.

[CR12] Bui T, Preuss CV (2022) Cephalosporins. In: StatPearls [Internet]. StatPearls Publishing, Treasure Island. https://www.ncbi.nlm.nih.gov/books/NBK551517/

[CR13] Bush K, Bradford PA (2016) Beta-lactams and beta-lactamase inhibitors: an overview. Cold Spring Harb Perspect Med 6(8). 10.1101/cshperspect.a025247 10.1101/cshperspect.a025247 PMC4968164 27329032

[CR14] Cabri W (2008). Industrial synthesis design with low environmental impact in the pharma industry. New methodologies and techniques for a sustainable organic chemistry.

[CR15] Calvo AM, Wilson RA, Bok JW, Keller NP (2002). Relationship between secondary metabolism and fungal development. Microbiol Mol Biol Rev.

[CR16] Caracuel Z, Roncero MIG, Espeso EA, González-Verdejo CI, García-Maceira FI, Di Pietro A (2003). The pH signalling transcription factor PacC controls virulence in the plant pathogen *Fusarium*
*oxysporum*. Mol Microbiol.

[CR17] Cassini A, Högberg LD, Plachouras D, Quattrocchi A, Hoxha A, Simonsen GS, Colomb-Cotinat M, Kretzschmar ME, Devleesschauwer B, Cecchini M (2019). Attributable deaths and disability-adjusted life-years caused by infections with antibiotic-resistant bacteria in the EU and the European Economic Area in 2015: a population-level modeling analysis. Lancet Infect Dis.

[CR18] Chen K, Chan EW-C, Xie M, Ye L, Dong N, Chen S (2017). Widespread distribution of mcr-1-bearing bacteria in the ecosystem, 2015 to 2016. Eurosurveillance.

[CR19] Cho KJ, Kim JK, Lee JH, Shin HJ, Park SS, Kim KH (2009). Structural features of cephalosporin acylase reveal the basis of autocatalytic activation. Biochem Biophys Res Commun.

[CR20] Cornelis P (2008). *Pseudomonas*: genomics and molecular biology.

[CR21] Dancer SJ (2001). The problem with cephalosporins. J Antimicrob Chemother.

[CR22] Domachowske J, Suryadevara M (2020). Chapter 58: Overview of antibiotics, 58.5 carbapenems. Clinical infectious diseases study guide: a problem-based approach.

[CR23] Dreyer J, Eichhorn H, Friedlin E, Kürnsteiner H, Kück U (2007). A homologue of the *Aspergillus* velvet gene regulates both cephalosporin C biosynthesis and hyphal fragmentation in *Acremonium*
*chrysogenum*. Appl Environ Microbiol.

[CR24] Drugs@FDA (2022) Center for Drug Evaluation and Research (U.S.). Food and Drug Administration. http://www.accessdata.fda.gov/scripts/cder/drugsatfda/ Accessed 03 March 2022

[CR25] Espeso EA, Tilburn J, Sánchez-Pulido L, Brown CV, Valencia A, Arst HN, Peñalva MA (1997). Specific DNA recognition by the *Aspergillus*
*nidulans* three zinc finger transcription factor PacC. J Mol Biol.

[CR26] Fleming A (1929). On the antibacterial action of cultures of a penicillium, with special reference to their use in the isolation of B. influenzæ. Br J Exp Pathol.

[CR27] Garau J, Wilson W, Wood M, Carlet J (1997). Fourth-generation cephalosporins: a review of in vitro activity, pharmacokinetics, pharmacodynamics and clinical utility. Clin Microbiol Infect.

[CR28] Gonzalez BE, Hulten KG, Dishop MK, Lamberth LB, Hammerman WA, Mason EO, Kaplan SL (2005). Pulmonary manifestations in children with invasive community-acquired *Staphylococcus*
*aureus* infection. Clin Infect Dis.

[CR29] Groeger H, Pieper M, Koenig B, Bayer T, Schleich H (2017). Industrial landmarks in the development of sustainable production processes for the beta-lactam antibiotic key intermediate 7-aminocephalosporanic acid (7-ACA). Sustain Chem Pharm.

[CR30] Harrison CJ, Bratcher D (2008). Cephalosporins: a review. Pediatr Rev.

[CR31] Hijarrubia M, Aparicio J, Martín J (2002). Nitrate regulation of α-aminoadipate reductase formation and lysine inhibition of its activity in *Penicillium*
*chrysogenum* and *Acremonium*
*chrysogenum*. Appl Microbiol Biotechnol.

[CR32] Hoff B, Schmitt EK, Kück U (2005). CPCR1, but not its interacting transcription factor AcFKH1, controls fungal arthrospore formation in *Acremonium*
*chrysogenum*. Mol Microbiol.

[CR33] Huber FM, Chauvette RR, Jackson BG (1972). Preparative methods for 7-aminocephalosporanic acid and 6-aminopenicillanic acid. Cephalosporins and penicillins: Chemistry and biology.

[CR34] Imada A, Kitano K, Kintaka K, Muroi M, Asai M (1981). Sulfazecin and isosulfazecin, novel beta-lactam antibiotics of bacterial origin. Nature.

[CR35] Jekosch K, Kück U (2000). Loss of glucose repression in an *Acremonium*
*chrysogenum* beta-lactam producer strain and its restoration by multiple copies of the *cre1* gene. Appl Microbiol Biotechnol.

[CR36] Kahan JS, Kahan FM, Goegelman R, Currie SA, Jackson M, Stapley EO, Miller TW, Miller AK, Hendlin D, Mochales S (1979). Thienamycin, a new beta-lactam antibiotic I. Discovery, taxonomy, isolation and physical properties. J Antibiot.

[CR37] Karaffa L, Sándor E, Kozma J, Szentirmai A (1996). Cephalosporin C production, morphology and alternative respiration of *Acremonium*
*chrysogenum* in glucose-limited chemostat. Biotechnol Lett.

[CR38] Karaffa L, Sándor E, Kozma J, Szentirmai A (1997). Methionine enhances sugar consumption, fragmentation, vacuolation and cephalosporin C production in *Acremonium*
*chrysogenum*. Process Biochem.

[CR39] Kim Y, Hol WG (2001). Structure of cephalosporin acylase in complex with glutaryl-7-aminocephalosporanic acid and glutarate: insight into the basis of its substrate specificity. Chem Biol.

[CR40] Kim S, Kim Y (2001). Active site residues of cephalosporin acylase are critical not only for enzymatic catalysis but also for post-translational modification. J Biol Chem.

[CR41] Kim Y, Yoon K, Khang Y, Turley S, Hol WG (2000). The 2.0 A crystal structure of cephalosporin acylase. Structure.

[CR42] Kim J, Ahn J (2022) Emergence and spread of antibiotic-resistant foodborne pathogens from farm to table. Food Sci Biotechnol:1–19. 10.1007/s10068-022-01157-1 10.1007/s10068-022-01157-1 PMC9435411 36065433

[CR43] Klein EY, Van Boeckel TP, Martinez Elena M, Pant S, Gandra S, Levin Simon A, Goossens H, Laxminarayan R (2018). Global increase and geographic convergence in antibiotic consumption between 2000 and 2015. PNAS.

[CR44] Laxminarayan R, Matsoso P, Pant S, Brower C, Røttingen J-A, Klugman K, Davies S (2016). Access to effective antimicrobials: a worldwide challenge. Lancet.

[CR45] Lee YS, Park SS (1998). Two-step autocatalytic processing of the glutaryl-7-aminocephalosporanic acid acylase from *Pseudomonas sp*. strain GK16. J Bacteriol.

[CR46] Li Y, Chen J, Jiang W, Mao X, Zhao G, Wang E (1999). In vivo post-translational processing and subunit reconstitution of cephalosporin acylase from *Pseudomonas sp*. 130. Eur J Biochem.

[CR47] Lin X, Lambertz J, Dahlmann TA, Nowaczyk MM, König B, Kück U (2022) A straightforward approach to synthesize 7-aminocephalosporanic acid in vivo in the cephalosporin C producer *Acremonium chrysogenum*. J Fungi 8(5). 10.3390/jof8050450 10.3390/jof8050450 PMC9144927 35628706

[CR48] Livermore DM (1987). Mechanisms of resistance to cephalosporin antibiotics. Drugs.

[CR49] Madison JH (1989). Eli Lilly, a Life, 1885–1977.

[CR50] Martínez-Martínez L (2008). Extended-spectrum beta-lactamases and the permeability barrier. Clin Microbiol Infect.

[CR51] Meyer H-P, Minas W, Schmidhalter D (2016). Industrial-scale fermentation. Industrial biotechnology: products and processes.

[CR52] Moellering RC, Eliopoulos GM, Sentochnik DE (1989). The carbapenems: new broad spectrum beta-lactam antibiotics. J Antimicrob Chemother.

[CR53] Moran GJ, Krishnadasan A, Gorwitz RJ, Fosheim GE, McDougal LK, Carey RB, Talan DA (2006). Methicillin-resistant *S. aureus* infections among patients in the emergency department. N Engl J Med.

[CR54] Morin RB, Roeske RW, Flynn EH, Jackson BG (1962). Chemistry of cephalosporin antibiotics. I. 7-Aminocephalosporanic acid from cephalosporin C. J Am Chem Soc.

[CR55] Murray CJL, Ikuta KS, Sharara F, Swetschinski L, Robles Aguilar G, Gray A, Han C, Bisignano C, Rao P, Wool E, Johnson SC, Browne AJ, Chipeta MG, Fell F, Hackett S, Haines-Woodhouse G, Kashef Hamadani BH, Kumaran EAP, McManigal B, Agarwal R, Akech S, Albertson S, Amuasi J, Andrews J, Aravkin A, Ashley E, Bailey F, Baker S, Basnyat B, Bekker A, Bender R, Bethou A, Bielicki J, Boonkasidecha S, Bukosia J, Carvalheiro C, Castañeda-Orjuela C, Chansamouth V, Chaurasia S, Chiurchiù S, Chowdhury F, Cook AJ, Cooper B, Cressey TR, Criollo-Mora E, Cunningham M, Darboe S, Day NPJ, De Luca M, Dokova K, Dramowski A, Dunachie SJ, Eckmanns T, Eibach D, Emami A, Feasey N, Fisher-Pearson N, Forrest K, Garrett D, Gastmeier P, Giref AZ, Greer RC, Gupta V, Haller S, Haselbeck A, Hay SI, Holm M, Hopkins S, Iregbu KC, Jacobs J, Jarovsky D, Javanmardi F, Khorana M, Kissoon N, Kobeissi E, Kostyanev T, Krapp F, Krumkamp R, Kumar A, Kyu HH, Lim C, Limmathurotsakul D, Loftus MJ, Lunn M, Ma J, Mturi N, Munera-Huertas T, Musicha P, Mussi-Pinhata MM, Nakamura T, Nanavati R, Nangia S, Newton P, Ngoun C, Novotney A, Nwakanma D, Obiero CW, Olivas-Martinez A, Olliaro P, Ooko E, Ortiz-Brizuela E, Peleg AY, Perrone C, Plakkal N, Ponce-de-Leon A, Raad M, Ramdin T, Riddell A, Roberts T, Robotham JV, Roca A, Rudd KE, Russell N, Schnall J, Scott JAG, Shivamallappa M, Sifuentes-Osornio J, Steenkeste N, Stewardson AJ, Stoeva T, Tasak N, Thaiprakong A, Thwaites G, Turner C, Turner P, van Doorn HR, Velaphi S, Vongpradith A, Vu H, Walsh T, Waner S, Wangrangsimakul T, Wozniak T, Zheng P, Sartorius B, Lopez AD, Stergachis A, Moore C, Dolecek C, Naghavi M (2022). Global burden of bacterial antimicrobial resistance in 2019: a systematic analysis. The Lancet.

[CR56] Navarro F (2006). Acquisition and horizontal diffusion of beta-lactam resistance among clinically relevant microorganisms. Int Microbiol.

[CR57] Newton GGF, Abraham EP (1954). Degradation, structure and some derivatives of cephalosporin N. Biochem J.

[CR58] Newton GG, Abraham EP (1955). Cephalosporin C, a new antibiotic containing sulphur and D-alpha-aminoadipic acid. Nature.

[CR59] Oh B, Kim M, Yoon J, Chung K, Shin Y, Lee D, Kim Y (2003). Deacylation activity of cephalosporin acylase to cephalosporin C is improved by changing the side-chain conformations of active-site residues. Biochem Biophys Res Commun.

[CR60] Otten LG, Sio CF, Van Der Sloot AM, Cool RH, Quax WJ (2004). Mutational analysis of a key residue in the substrate specificity of a cephalosporin acylase. ChemBioChem.

[CR61] Papp-Wallace KM, Endimiani A, Taracila MA, Bonomo RA (2011). Carbapenems: past, present, and future. Antimicrob Agents Chemother.

[CR62] Paterson DL, Bonomo RA (2005). Extended-spectrum beta-lactamases: a clinical update. Clin Microbiol Rev.

[CR63] Pichichero ME, Casey JR (2007). Safe use of selected cephalosporins in penicillin-allergic patients: a meta-analysis. Otolaryngol Head Neck Surg.

[CR64] Pollegioni L, Rosini E, Molla G (2013). Cephalosporin C acylase: dream and(/or) reality. Appl Microbiol Biotechnol.

[CR65] Poole K (2004). Efflux-mediated multiresistance in Gram-negative bacteria. Clin Microbiol Infect.

[CR66] Poole K (2007). Efflux pumps as antimicrobial resistance mechanisms. Ann Med.

[CR67] Raj GM (2021). Penicillins, cephalosporins and other beta-lactam anticiotics. Introduction to basics of pharmacology and toxicology, Vol 2: Essentials of systemic pharmacology : from principles to practice.

[CR68] Riethorst W, Reichert A (1999). An industrial view on enzymes for the cleavage of cephalosporin C. Chimia.

[CR69] Sándor E, Pusztahelyi T, Karaffa L, Karányi Z, Pócsi I, Biró S, Szentirmai A, Pócsi I (1998). Allosamidin inhibits the fragmentation of *Acremonium*
*chrysogenum* but does not influence the cephalosporin C production of the fungus. FEMS Microbiol Lett.

[CR70] Schmitt EK, Kempken R, Kück U (2001). Functional analysis of promoter sequences of cephalosporin C biosynthesis genes from *Acremonium*
*chrysogenum*: specific DNA-protein interactions and characterization of the transcription factor PACC. Mol Genet Genom.

[CR71] Schmitt EK, Bunse A, Janus D, Hoff B, Friedlin E, Kürnsteiner H, Kück U (2004). Winged helix transcription factor CPCR1 is involved in regulation of beta-lactam biosynthesis in the fungus *Acremonium*
*chrysogenum*. Eukaryot Cell.

[CR72] Schmitt EK, Hoff B, Kück U (2004). Regulation of cephalosporin biosynthesis. Molecular biotechnology of fungal beta-lactam antibiotics and related peptide synthetases, Vol 88, 2004.

[CR73] Selvan SR, Ganapathy D (2016). Efficacy of fifth-generation cephalosporins against methicillin-resistant *Staphylococcus*
*aureus*-a review. Res J Pharm Technol.

[CR74] Silver LL (2011). Challenges of antibacterial discovery. Clin Microbiol Rev.

[CR75] Suárez T, Peñalva MA (1996). Characterization of a *Penicillium*
*chrysogenum* gene encoding a PacC transcription factor and its binding sites in the divergent *pcbAB–pcbC* promoter of the penicillin biosynthetic cluster. Mol Microbiol.

[CR76] Sullins AK, Abdel-Rahman SM (2013). Pharmacokinetics of antibacterial agents in the CSF of children and adolescents. Paediatr Drugs.

[CR77] Sykes RB, Cimarusti CM, Bonner DP, Bush K, Floyd DM, Georgopapadakou NH, Koster WH, Liu WC, Parker WL, Principe PA (1981). Monocyclic beta-lactam antibiotics produced by bacteria. Nature.

[CR78] Tacconelli E, Carrara E, Savoldi A, Harbarth S, Mendelson M, Monnet DL, Pulcini C, Kahlmeter G, Kluytmans J, Carmeli Y (2018). Discovery, research, and development of new antibiotics: the WHO priority list of antibiotic-resistant bacteria and tuberculosis. Lancet Infect Dis.

[CR79] Tan Q, Qiu J, Luo X, Zhang Y, Liu Y, Chen Y, Yuan J, Liao W (2018). Progress in one-pot bioconversion of cephalosporin C to 7-aminocephalosporanic acid. Curr Pharm Biotechnol.

[CR80] Tartaglione TA, Polk RE (1985). Review of the new second-generation cephalosporins: cefonicid, ceforanide, and cefuroxime. Drug Intell Clin Pharm.

[CR81] Terfehr D, Dahlmann TA, Kück U (2017). Transcriptome analysis of the two unrelated fungal beta-lactam producers *Acremonium*
*chrysogenum* and *Penicillium*
*chrysogenum*: velvet-regulated genes are major targets during conventional strain improvement programs. BMC Genomics.

[CR82] Terreni M, Taccani M, Pregnolato M (2021). New antibiotics for multidrug-resistant bacterial strains: latest research developments and future perspectives. Molecules.

[CR83] Tipper DJ, Strominger JL (1965). Mechanism of action of penicillins: a proposal based on their structural similarity to acyl-D-alanyl-D-alanine. PNAS.

[CR84] Ullan RV, Liu G, Casqueiro J, Gutierrez S, Banuelos O, Martin JF (2002). The *cefT* gene of *Acremonium*
*chrysogenum* C10 encodes a putative multidrug efflux pump protein that significantly increases cephalosporin C production. Mol Genet Genomics.

[CR85] Velasco J, Gutierrez S, Fernandez F, Marcos A, Arenos C, Martin J (1994). Exogenous methionine increases levels of mRNAs transcribed from *pcbAB*, *pcbC*, and *cefEF* genes, encoding enzymes of the cephalosporin biosynthetic pathway *Acremonium*
*Chrysogenum*. J Bacteriol.

[CR86] Versporten A, Bruyndonckx R, Adriaenssens N, Hens N, Monnet DL, Molenberghs G, Goossens H, Weist K, Coenen S (2021). Consumption of cephalosporins in the community, European Union/European Economic Area, 1997–2017. J Antimicrob Chemother.

[CR87] Wilson WR (1998). The role of fourth-generation cephalosporins in the treatment of serious infectious diseases in hospitalized patients. Diagn Microbiol Infect Dis.

[CR88] Wohlgemuth R (2010). Large-scale applications of hydrolases in biocatalytic asymmetric synthesis. Asymmetric catalysis on industrial scale.

[CR89] Zhanel GG, Sniezek G, Schweizer F, Zelenitsky S, Lagace-Wiens PR, Rubinstein E, Gin AS, Hoban DJ, Karlowsky JA (2009). Ceftaroline: a novel broad-spectrum cephalosporin with activity against methicillin-resistant *Staphylococcus*
*aureus*. Drugs.

[CR90] Zhanel GG, Lawson CD, Adam H, Schweizer F, Zelenitsky S, Lagacé-Wiens PRS, Denisuik A, Rubinstein E, Gin AS, Hoban DJ, Lynch JP, Karlowsky JA (2013). Ceftazidime-avibactam: a novel cephalosporin/beta-lactamase inhibitor combination. Drugs.

[CR91] Zhanel GG, Golden AR, Zelenitsky S, Wiebe K, Lawrence CK, Adam HJ, Idowu T, Domalaon R, Schweizer F, Zhanel MA, Lagace-Wiens PRS, Walkty AJ, Noreddin A, Lynch Iii JP, Karlowsky JA (2019). Cefiderocol: a siderophore cephalosporin with activity against carbapenem-resistant and multidrug-resistant Gram-negative bacilli. Drugs.

[CR92] Zhang J, Demain AL (1992). Regulation of ACV synthetase activity in the beta-lactam biosynthetic pathway by carbon sources and their metabolites. Arch Microbiol.

[CR93] Zhang J, Wolfe S, Demain AL (1989). Carbon source regulation of ACV synthetase in *Cephalosporium*
*acremonium* C10. Curr Microbiol.

[CR94] Zhgun A, Dumina M, Valiakhmetov A, Eldarov M (2020). The critical role of plasma membrane H^+^-ATPase activity in cephalosporin C biosynthesis of *Acremonium*
*chrysogenum*. PLoS ONE.

[CR95] Zhu X, Luo H, Chang Y, Su H, Li Q, Yu H, Shen Z (2011). Characteristic of immobilized cephalosporin C acylase and its application in one-step enzymatic conversion of cephalosporin C to 7-aminocephalosporanic acid. World J Microbiol Biotechnol.

